# Supplementary figures and images for: HIV-1 controllers possess a unique CD8+ T cell activation phenotype and loss of control is associated with increased expression of exhaustion markers
Source: PLoS One. 2025 Aug 28;20(8):e0328706. doi: 10.1371/journal.pone.0328706 (PMC12393755; doi:10.1371/journal.pone.0328706)

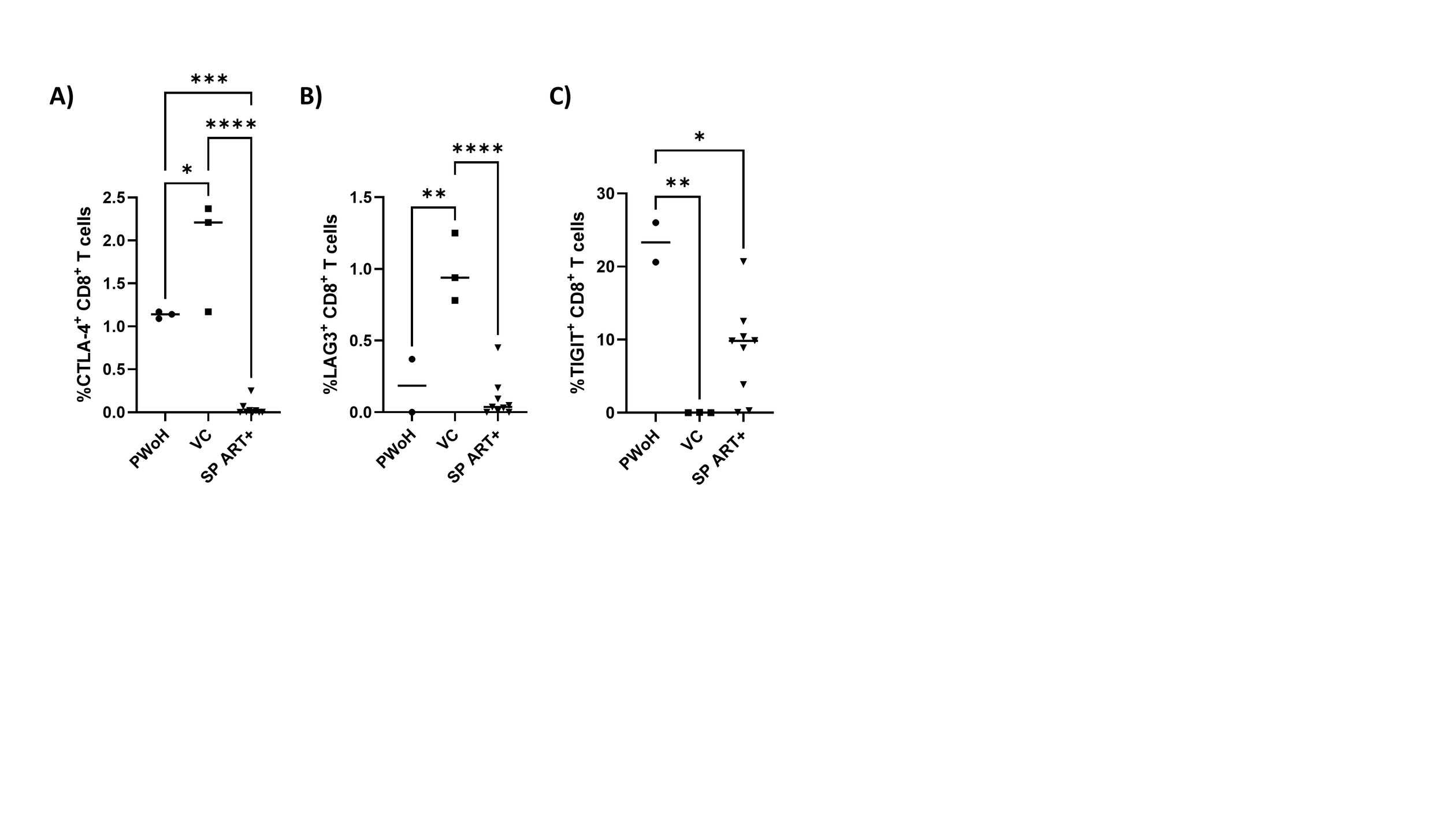

Supplement: S1 Fig — PBMCs were isolated from blood samples of PWoH (n=2-3), VC (n=3) and SP ART- (n=9) and flow cytometry was performed gating for live (Zombie Yellow-), CD8+ T cells (CD3+CD8+). Graphed are the percentages of live CD8+ T cells that are A) CTLA-4+, B) LAG3+, or C) TIGIT+. Ordinary one-way ANOVA with Tukey’s multiple comparisons test was performed to determine statistical significance. * p≤0.05, ** p≤0.01, *** p≤0.0005, **** p≤0.0001. (TIF) [file pone.0328706.s001.tif]

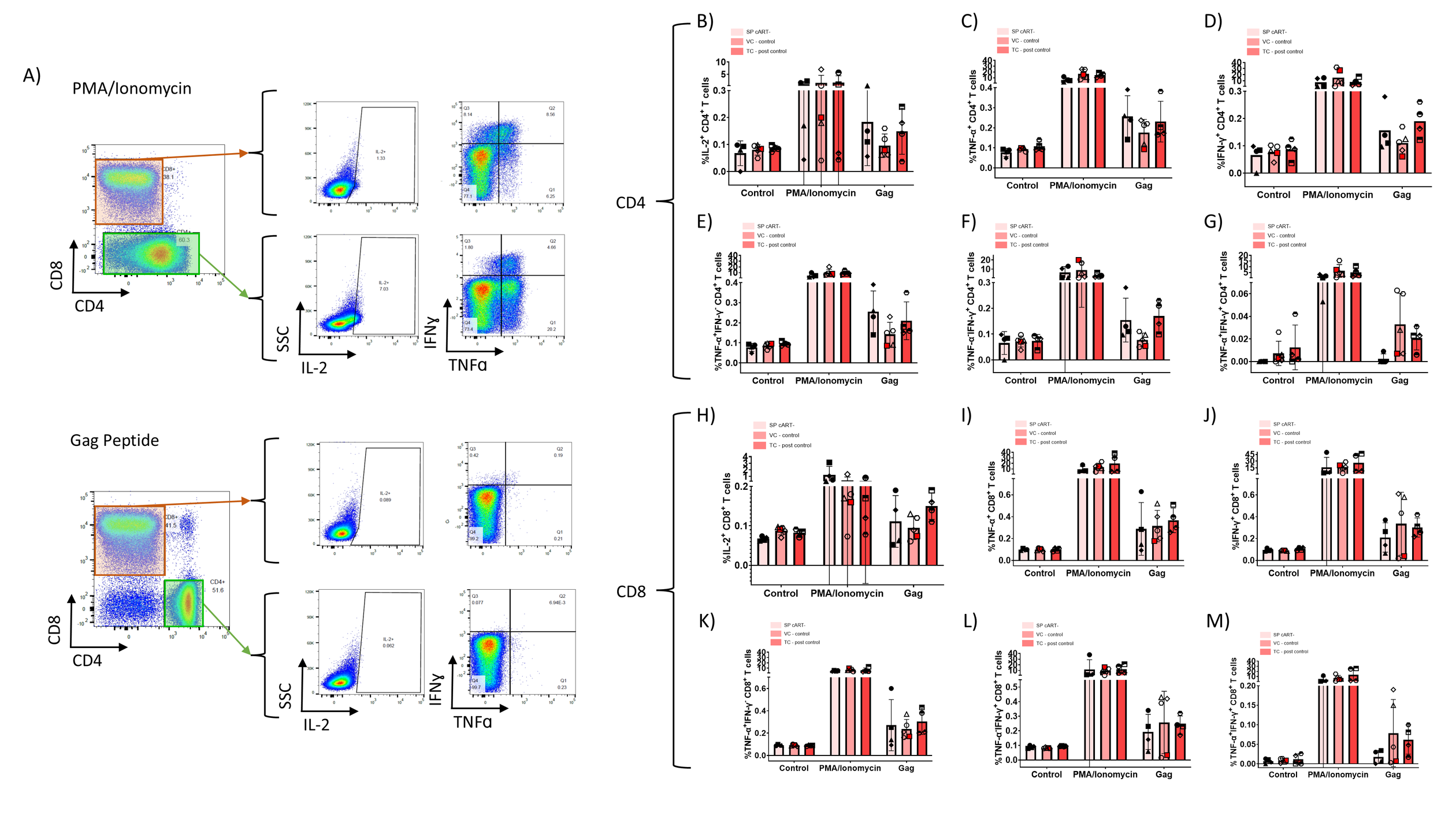

Supplement: S2 Fig — PBMCs from SP cART- (N=4), VC - control (N=5), and TC – post control (N=4) were unstimulated (control), stimulated with PMA/Ionomycin (50ng/mL and 500ng/mL, respectively) or Gag peptide pool (2μg/mL/peptide) in the presence of co-stimulatory antibodies CD28/CD49d (1μg/mL/each) for 1 h, followed by treatment with GolgiPlug for 4.5 h. Flow cytometry was performed gating for live (Zombie Yellow), CD4+ T cells (CD3+CD4+) or CD8+ T cells (CD3+CD8+) followed by cytokines IL-2, TNF-ɑ, and IFN-ɣ. A) Representative gating of PMA/Ionomycin and Gag peptide stimulated CD4+ and CD8+ T cells analyzed for the expression of IL-2, TNF-ɑ, and IFN-ɣ (Donor SRS5930, VC - control). Percentage of live CD4+ T cells that are B) IL-2+, C) TNF-ɑ+ (IFN-ɣ+TNF-ɑ+ + IFN-ɣ-TNF-ɑ+), D) IFN-ɣ+ (IFN-ɣ+TNF-ɑ+ + IFN-ɣ+TNF-ɑ-), E) TNF-ɑ+IFN-ɣ-, F) TNF-ɑ-IFN-ɣ+, G) TNF-ɑ+IFN-ɣ+ are quantified for control, PMA/Ionomycin, or Gag peptide treated cells. Percentage of live CD8+ T cells that are H) IL-2+, I) TNF-ɑ+ (IFN-ɣ+TNF-ɑ+ + IFN-ɣ-TNF-ɑ+), J) IFN-ɣ+ (IFN-ɣ+TNF-ɑ+ + IFN-ɣ+TNF-ɑ-), K) TNF-ɑ+IFN-ɣ-, L) TNF-ɑ-IFN-ɣ+, M) TNF-ɑ+IFN-ɣ+ are quantified for control, PMA/Ionomycin, or Gag peptide treated cells. Gates were set based on the control population (CD28/CD49d treated only) such that the positive population is less than 0.1%. Data shown as means ± SD. Ordinary one-way ANOVA with Tukey’s multiple comparisons test was performed to determine statistical significance within donor classifications. (TIF) [file pone.0328706.s002.tif]

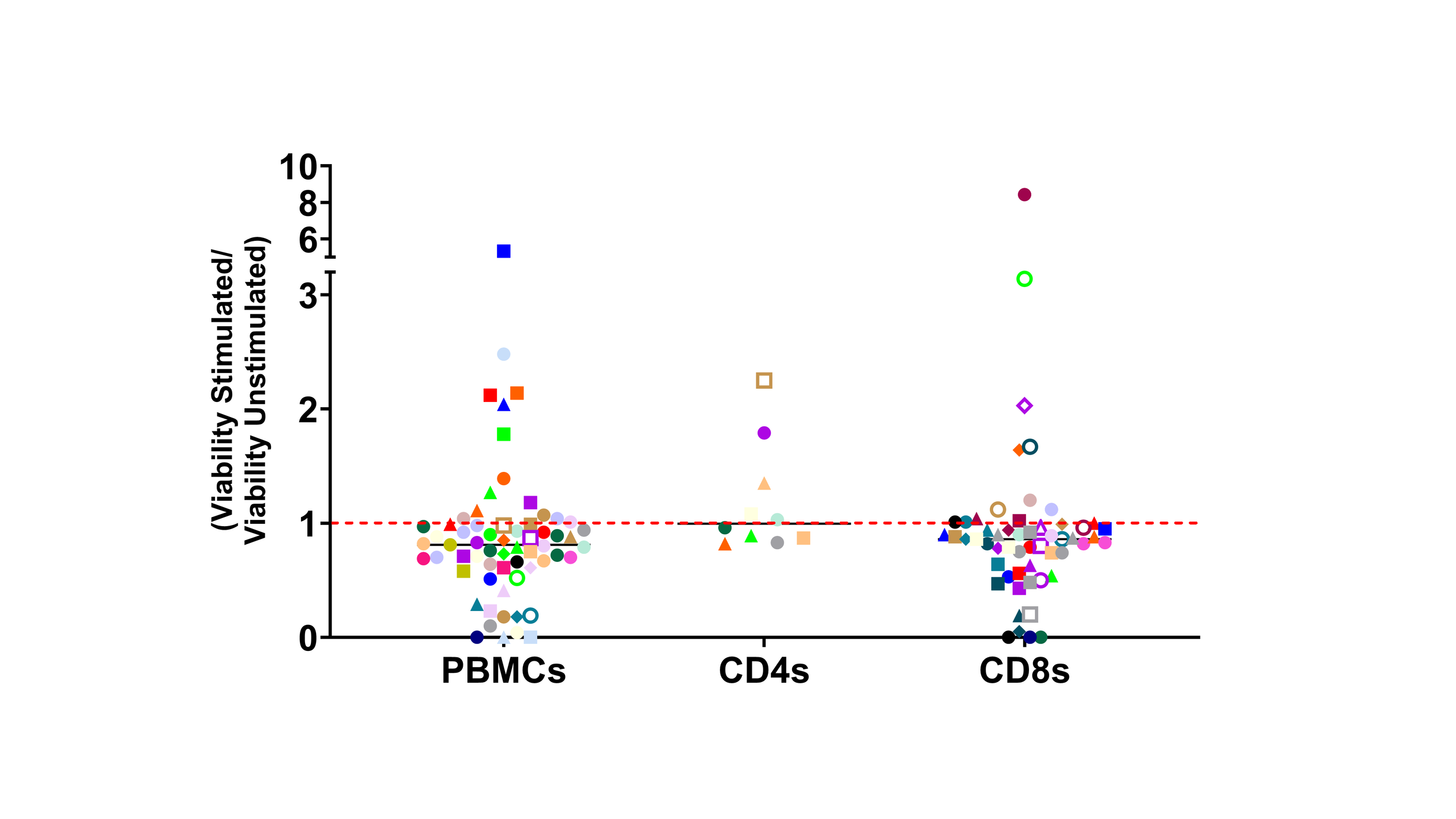

Supplement: S3 Fig — PBMCs (n=63), CD4+ T cell isolates (n=10), and CD8+ T cell isolates (n=55) were stimulated with PHA-P (5 μg/mL) in the presence of human rIL-2 (5 U/mL) for 48 h. Cells were subsequently washed, collected, and counted; live cells were determined by the absence of trypan blue stain. Change in viability (Δ Viability) was calculated as the ratio of the percent viability of stimulated cells (live stimulated count/total stimulated count) to the percent viability of unstimulated (live unstimulated count/total unstimulated count) cells. Symbol colors represent individual donors and symbol shapes represent individual blood draw dates. Ordinary one-way ANOVA with Tukey’s multiple comparisons test was performed to determine statistical significance. (TIF) [file pone.0328706.s003.tif]
