# Supplementary material for: HIV-1 controllers possess a unique CD8+ T cell activation phenotype and loss of control is associated with increased expression of exhaustion markers
Source: PLoS One. 2025 Aug 28;20(8):e0328706. doi: 10.1371/journal.pone.0328706 (PMC12393755; doi:10.1371/journal.pone.0328706)
Supplement: Attachment — Submitted filename: Response to Reviewers.docx [file pone.0328706.s006.docx]

We would like to thank the reviewer for their time and effort in critiquing our manuscript. We have been able to address all of the presented comments and believe that these changes much improve the work. In response to reviewer comments (noted below) we have updated the nomenclature used to describe our different study participant categories: standard progressor (SP ART- or ART+), virologic controller (VC) – including two subgroups of persistent (VC) and transient (TC) controllers. We use these category labels in the reviewer response. In updating the labels we noted that figure 3 (examination of CTLA4, LAG3 and TIGIT) compares PWoH to VC to SP ART+, unlike figures 1 and 2 that compare to SP ART-. As this is not the same comparison, we have de-emphasized this data by moving it into the supplemental figures.

Responses to reviewer’s points appear in *red*.

Section 1:

There is great plasticity in CD8+ T cells (central, effector, etc) but here, the granularity is lost since bulk CD8s are examined. So while exhaustion markers are used, functionality of the CD8s is the other half that seems to be missing. The study does well in providing an overall sense of basal levels but there is much more that could and probably should be explored here. It would have been nice to add HIV peptide stimulation (with controls) to further tease out how exhaustion and cytolytic function are entwined.

*We agree with the reviewer that correlating observed CD8^+^ exhaustion profiles reported in this manuscript and our previous publication [19] with CD8 functionality will be important to further classify clinical phenotypes of the donors. To address this, we measured cytokine production from PBMCs from the following donor groups: standard progressors in the absence of ART (SP cART-), viremic controllers (VC - control) during periods of successful control, and transient controllers after the loss of control (TC – post control).  We performed an intracellular cytokine assay (ICC) where PBMCs from each donor group were unstimulated (control), stimulated with PMA/Ionomycin, or with Gag peptide pool in the presence of co-stimulation with anti-CD28/CD49d antibodies for 1 hr, then treated with GolgiPlug (Brefeldin A) to prevent secretion of cytokines, resulting in the accumulation of cytokines intracellularly which can then be detected by flow cytometry.  We measured the production of IL-2, TNF-ɑ, and IFN-ɣ from CD4^+^ T cells and CD8^+^ T cells, and despite measuring general and HIV-specific cytokine accumulation, we did not observe any statistically significant changes in cytokine production from either cell type when comparing responses within donor groups.  These data indicate that there does not appear to be a correlation between cytokine production from HIV-responsive cells, CD8^+^ T cell exhaustion, and clinical classification of donors based on presence of control or ART. In the future, we hope to be able to assess the effects of CD8^+^ T cell exhaustion on cytolytic activity, specifically granzyme B production and cell killing. Additionally, using pooled Gag peptides to stimulate cells did not allow us to determine Gag epitope specificity in the context of HIV-1 controllers. We present this data in the updated manuscript as Supplemental Figure 2 and include relevant changes to Materials and Methods, Results, and Discussion. Our original publication demonstrated the need for cell-cell contact in mediating viral suppression in these donors. This suggests that changes may be seen when analyzing cell killing, which we did not do in this work. As for the limitation in granularity due to analyzing bulk CD8s, we agree and plan to assess differences in CD8^+^ T cell subsets in the future. Due to the scarcity of samples available to us from this cohort, we were limited to assessing bulk CD8 T cells. To address this concern, we have included some verbiage in the discussion pointing out this limitation to the reader.*

Given the limitation in the number of participants, it is a worry on the Power to know if the differences between "high" vs. "low" levels of plasma viremia can be parsed out--clarification on whether the "low" are actually viremic controllers is needed--with the inclusion of one Elite controller is nice to have but would be nice if it were at least three. ->

*We agree with the reviewer that the number of participants is a limitation of the study and have included reference to this fact as a limitation in the discussion section. The categories were originally based on observation of clinical outcomes by our partnering physician – evidence for maintained low level of plasma viremia. This is discussed in more detail in Jones et al 2021 reference [19] in this publication. Here is the analysis of plasma viraemia and CD4+ t-cell counts of these donors as originally observed:*

*We have also updated the participant group names to hopefully provide more clarity on the classification of viremic, persistent, transient, and elite controllers. The new participant group names are described in the methods section and now appear throughout the text, figures/figure legends, and tables 1, 2, and 3. We also provided clinical criteria for how donors were considered viremic vs. elite controllers in the “Study cohort” section of the methods. Of note, the elite controller included here and the other from the original study had undetectable viral loads (as opposed to the detectable viremia in our controllers) and did not demonstrate any control of de novo infection of their PBMCs.*

One of the biggest challenges came from the writing. The methods need to be fleshed out more. -> *We agree with the reviewer and apologize for our lack of details and clarity in the original methods section. To address this concern, we have added more details to the methods, highlighted for each section below:*

- *Study cohort: added more clarification on how donors were classified and explained new group naming system.*
- *PBMC isolation and activation: included more detail on the PBMC isolation protocol including centrifugation speeds, times, and temperatures, as well as more details on specific reagents used.*
- *CD4^+^ and CD8^+^ T cell isolation and activation: included more details on specific reagents used and protocol steps leading to downstream experiments.*
- *HIV-1 virus stock: included more details on specific reagents used and more details on the specific protocol used to isolate, filter, and store the virus stocks. Also included explanation on how virus stocks were titered and how the specific MOI used throughout the study was determined.*
- *Immune checkpoint blockade viral suppression assay: included details on more specific steps of the protocol and more details on specific reagents used. Also included the method for calculating % suppression of viral replication.*
- *Flow cytometry: included more detail on specific incubation and wash steps (time, temperature, etc.) as well as more specific details on reagents used.*
- *Multivariate clustering analysis: clarified some wording.*
- *Statistics: provided more details on graphing for specific figures, and updated figure legends to include more specific statistical analysis details rather than including in the statistics section.*

The introduction had "pacing" issues. ->

*We agree with the reviewer and have made changes to clarify the narrative presented in the introduction. We have merged the first two paragraphs and cut out details that were not entirely necessary to understand the background of this study. We also added more relevant references and provided more context for this study based on findings from our previous study.*

If possible for the results, either combine sections to take more time providing results.

State what p-values might be instead of just relying on the legends.

There were several cases where not all of the figure panels were discussed either.

*We have now included more details in the results sections including: adding references to contextualize findings and have attempted to add more interpretation of the findings. P values are now present throughout the text wherever a statistically significant or non-significant result is mentioned. All figure panels should now be referenced in the text.*

Data visualization (minus the tables), was very clear (font in Fig 7 could be increased). ->

*We agree with the reviewer that the tables could have been clearer, so we redesigned them for clarity and ease of reading and updated them with the improved group naming scheme. We also increased the font in figure 7.*

Section 2:

Lines 41-57: It is commendable that the authors are trying to compact HIV clinical progression with many factors that are at play. It should be tightened and explore the space. For instance, “HIV-1 without therapy”, those familiar will know this as ART, but it would benefit to say “HIV-1 without antiretroviral therapy (ART)”. Please also be conscious to use People First language, “patient” is no longer accepted, but study participant or people/persons with HIV (PWH)—Please also see Methods as PWH are not “subjects”. The second paragraph suffers from much of the same issues as the first. It is clear the authors are being thoughtful, but this Reviewer would strongly suggest making more succinct. ->

*Thank you, we agree on all points. We have removed details that were not totally necessary and merged the first two paragraphs to improve the flow of the writing. We also updated the text to use "ART" instead of “therapy” and defined it at first usage. The text was also updated to include people first language (“patient”, ”subject”, and “donor” were changed to “study participant” and “normal donor” was changed to “people without HIV (PWoH)”.*

Lines 94-96: The authors do a nice job discussing Tim-3 and PD-1 but then just list other markers. Perhaps in condensing the first two paragraphs, this paragraph can be expanded. ->

*We agree with the reviewer that the introduction of the other markers was rushed so we balanced the information on the various exhaustion markers in the introduction and added more information about them as they appear in the results sections.*

Line 130: Since MHC-II HLA alleles are known, suggest including in Table 1. ->

*We agree with the reviewer, so we created a new table (Table 2) which includes MHC-II allele information for the majority of the donors.*

Line 136: Are the LVL donors viremic controllers? Based on the cut-offs it appears so but not sure if that is your aim. ->

*We recognize that this was confusing in the original manuscript, so we have changed the naming scheme of the groups to hopefully be clearer. The “LVL” donors referred to in the original manuscript are viremic controllers and this is now clearer in the methods section as well as table 1. Also, criteria for being considered a viremic controller is included in the "Study cohort" section in the methods.*

Line 143: The authors have copies/ml and copies/mL. It is not clear why for one donor “ND” was used for CD8 count but Persons without HIV “Pw/oH” just have a dash. Some numbers have commas others do not. Cell counts need the mm-cubed to actually be a superscript. For ART regimens, it is better to spell out the non-brand name (Truvada=TDF/FTC=tenofovir/emtricitabine). ->

*We have improved table 1 regarding the errors identified here and have included a key for table 1 that explains all the symbols used in the table (and have taken similar steps with the new table 2). Specifically, we updated “copies/ml” to “copies/mL”, removed “ND” and replaced with “-“ indicating the data was not available, updated numbers to include commas consistently, updated “mm3” to “mm^3^”, and updated cART regimens to display the non-brand drug names.*

Line 151: Out of curiosity, what was cell viability for a 48hr PHA stimulation? Typically 24hrs is more than enough before it starts to become toxic. ->

*We agree with the reviewer that cell viability can be affected by PHA-P stimulation.  We monitor viability in all of our experiments and generally exclude material that either starts with low viability or dies during the experiment. We now include Supplemental Figure 3 where we have graphed the effects of PHA treatment on viability of PBMCs, CD4 isolates, and CD8 isolates collated from an expansive collection of donors from the cohort used throughout this and other studies.  The ratio of viability was calculated based on the presence or absence of trypan blue stain as the ratio of the percent viability of stimulated cells (live stimulated count/total stimulated count) to the percent viability of unstimulated (live unstimulated count/total unstimulated count). Induction of cells with PHA-P resulted in a minimal change in viability compared to unstimulated cells (PBMCs – 0.89, CD4^+^- 1.187, and CD8^+^ - 0.98). These data indicate that on average, there is no appreciable effect of PHA-P stimulation on cell viability after 48hr in our hands. Appropriate updates to the methods and results section are now included to reference inclusion of this data.*

Lines 162-163: More information is needed for virus collection. How was the virus “filtered”. Frozen stocks at what temperature? ->

*We agree with the reviewer that more details should have been included for virus collection. We have updated the methods section to include the details pointed out and more. Specifically, virus was filtered to exclude cells with a 0.22 µm filter and 1 mL aliquots were frozen in liquid nitrogen.*

Line 170: 17ng/ml of NL4-3 is oddly very specific. How was this amount of virus determined for the infection? It would be nice to also know what the MOI was considered. ->

*Cultures were infected at an MOI = 0.01 and for this particular viral stock and culture conditions that equated to the mentioned 17ng/ml. We have updated language in the text to use MOI instead of ng/mL for clarity, and included details on how this MOI was determined in the “HIV-1 virus stock” section of the methods.*
