# Supplementary material for: HIV-1 controllers possess a unique CD8+ T cell activation phenotype and loss of control is associated with increased expression of exhaustion markers
Source: PLoS One. 2025 Aug 28;20(8):e0328706. doi: 10.1371/journal.pone.0328706 (PMC12393755; doi:10.1371/journal.pone.0328706)
Supplement: Attachment — Submitted filename: Response to Reviewers - 2nd sub.docx [file pone.0328706.s007.docx]

We would like to thank the reviewer for their time and effort in critiquing our manuscript. We have been able to address the newly presented comments and believe that these changes much improve the work.

Responses to reviewer’s points appear in *red*.

Reviewer #1: The revised manuscript by Jones and Capriotti et al. is very much improved from the original draft. It is greatly appreciated the time the authors took to address comments and rework many aspects. It is a much stronger and compelling body of work. The only minor suggestion is with p-values, sometimes the number of sig. figs is too much (e.g., p=0.0402 could just be 0.04 but p=0.0003 is 0.0003). They are not inherently wrong, but I am not sure the sample size is large enough to go to that many sig. figs.

*We agree with the reviewer that the amount of significant figures included in the reporting of p values may have been too high in the revised manuscript. To address this, we have rounded all p values reported in the manuscript to include just one significant figure.*
